# Difference in Strength Development between Cement-Treated Sand and Mortar with Various Cement Types and Curing Temperatures

**DOI:** 10.3390/ma13214999

**Published:** 2020-11-06

**Authors:** Lanh Si Ho, Kenichiro Nakarai, Kenta Eguchi, Yuko Ogawa

**Affiliations:** 1 Civil and Environmental Engineering Program, Graduate School of Advanced Science and Engineering, Hiroshima University, Higashihiroshima 739-8527, Japan; lanhhs@utt.edu.vn (L.S.H.); nakarai@hiroshima-u.ac.jp (K.N.); 2 Department of Civil and Environmental Engineering, Graduate School of Engineering, Hiroshima University, Higashihiroshima 739-8527, Japan; k_eguchi@shimz.co.jp

**Keywords:** cement-treated soil, mortar, high early-strength Portland cement, chemically bound water, thermal analysis, elevated temperature

## Abstract

To improve the strength of cement-treated sand effectively, the use of various cement types was investigated at different curing temperatures and compared with the results obtained from similar mortars at higher cement contents. The compressive strengths of cement-treated sand specimens that contained high early-strength Portland cement (HPC) cured at elevated and normal temperatures were found to be higher than those of specimens that contained ordinary Portland cement (OPC) and moderate heat Portland cement at both early and later ages. At 3 days, the compressive strength of the HPC-treated sand specimen, normalized with respect to that of the OPC under normal conditions, is nearly twice the corresponding value for the HPC mortar specimens with water-to-cement ratio of 50%. At 28 days, the normalized value for HPC-treated sand is approximately 1.5 times higher than that of mortar, with a value of 50%. This indicates that the use of HPC contributed more to the strength development of the cement-treated sand than to that of the mortar, and the effects of HPC at an early age were higher than those at a later age. These trends were explained by the larger quantity of chemically bound water observed in the specimens that contained HPC, as a result of their greater alite contents and porosities, in cement-treated sand. The findings of this study can be used to ensure the desired strength development of cement-treated soils by considering both the curing temperature and cement type. Furthermore, they suggested a novel method for producing a high internal temperature for promoting the strength development of cement-treated soils.

## 1. Introduction

Cement-treated soils are extensively used for ground improvement, especially in deep-mixing treatments. The short-term strength of a cement-treated soil is a result of cement hydration, and its long-term strength is a result of the pozzolanic reaction between the cement hydration products and soil particles [1,2,3,4]. These chemical reactions can be affected by elevated temperatures caused by internal or external factors. For example, in the deep-mixing treatment method, a large cement-treated soil column is employed in which the heat of the hydration can lead to relatively high temperatures of ~50 °C in its core, which remain constant for several months [5]. Furthermore, in tropical regions, the temperature recorded within cement-treated clay can reach 38 °C [6]. These high-temperature conditions can accelerate the chemical reactions in cement-treated soil, including the cement hydration and pozzolanic reactions, and can thus result in an enhanced compressive strength. Several researchers have observed that the strength evolution of cement-treated clays is promoted in both the short and long terms (3 and 91 days of curing, respectively) at high-curing temperatures in the range of 40–60 °C [6,7,8,9]. Additionally, scanning electron microscopic analyses have detected new cementitious components, such as C-S-H and C-A-H, in cement-treated clays cured at high temperatures [6,7,8,9,10,11]. These additional formations pertaining to the cementitious phases lead to an increase in the material strength, and represent microstructural changes owing to the occurrence of cement hydration and pozzolanic reactions at high temperatures. These changes can be quite beneficial in practical applications, as they help improve the short- and long-term stabilities of cement-treated soils [12]. However, to date, few studies have been published that investigate the relationship between the curing temperature and strength of the cement-treated soils with respect to various material parameters.

However, many studies have conducted detailed investigations on the effects of the curing temperature on conventional cement concrete, and have observed different behaviors at elevated temperatures compared with cement-treated soils. At an early age, the compressive strength development of concrete is promoted at elevated curing temperatures owing to the accelerated hydration process [13,14,15]. However, the increment rate of the compressive strength over long-term curing (i.e., 91 days) decreases with an increasing curing temperature (from 20 to 60 °C). In particular, high curing temperatures cause cement hydration products to be arranged in a nonuniform manner, thus creating large pores in a phenomenon known as the cross-over effect [15,16,17,18]. Furthermore, the effects of temperature on concrete are strongly dependent on the type of cement employed. It has been reported that the use of blended cement containing supplementary cementitious materials, such as fly ash, slag, or silica fume, can enhance the long-term strengths of concrete or mortar exposed to high temperatures, especially in the range of 35–50 °C [19]. Specifically, the pozzolanic reaction is promoted at elevated temperatures, thus generating additional C-S-H phases that reduce the cross-over effect [20]. Consequently, the compressive strengths of these blended cements increase at both early and later ages with high-temperature curing. Monzo et al. [21] examined fly ash-blended cement, and found that the optimal curing temperature for promoting the pozzolanic reaction was 40 °C. Moreover, previous studies have noted that the compressive strengths of blended cements that contained volcanic ash increased at increasing curing temperatures between 20 and 40 °C [13,22]. In addition, recent studies have reported the temperature-dependent properties of geopolymer mortar as a new construction material. For example, some previous studies found that higher curing temperatures could also enhance the strength evolution of geopolymer mortar [23,24,25].

Many published studies have also discussed the effects of temperature on concrete that contains various types of cement. High early-strength Portland cement (HPC) with high alite content is commonly used in concrete and mortar to promote the cement hydration process. In general, the activation energy and temperature attained from the use of HPC are larger than those generated during the use of ordinary Portland cement (OPC) [26]. Thus, the early-age compressive strengths of specimens that contain HPC are typically higher. In contrast, the use of moderate heat Portland cement (MPC) with high belite contents provides a lower strength at an early age, but enables strength development at a later age [27]. In a previous study that investigated the characteristics of high-strength concrete that contained MPC, it was observed that after 91 days of curing at elevated temperatures (e.g., at 38 and 70 °C), the strength of MPC concrete was greater than that of OPC concrete [28].

The results of previous research thus indicate that elevated temperatures can be applied to accelerate the strength development of cement-treated soils. However, this approach is subject to certain limitations in practical applications. In general, elevated curing temperatures are generated in a laboratory environment via external heating with a control temperature chamber or a hot water bath. These methods are expensive and difficult to apply on real construction sites. However, as previously mentioned, HPC can generate a high internal temperature owing to its high-activation energy. Thus, it is likely that HPC could be employed in a novel approach to realize a high internal curing temperature that activates chemical reactions at an early age, thus accelerating strength development. To apply this approach to cement-treated soils and examine the resulting strength development, two main factors must be considered: cement type and curing temperature.

Accordingly, the objective of this study was to evaluate the strength evolution of HPC-treated sand at different curing temperatures, to obtain basic information for the development of a novel method to produce a high internal temperature for promoting the strength development of cement-treated soils. To understand the behavior of the HPC-treated sand, the use of MPC and OPC were also examined for reference. Moreover, the cement content, as assessed by evaluating mortar specimens that contained a large quantity of cement, was also considered as a parameter. One of the novel aspects of this study is that the approach used connects cement-treated sand, which is intensively studied in the geotechnical field, and mortar, which is widely used in the concrete field.

The compressive strengths of cement-treated sand and mortar specimens were first determined with the use of unconfined compression tests to examine the changes in their mechanical properties according to the cement type and curing temperature. The chemicophysical properties were then investigated by performing a thermogravimetric-differential thermal analysis (TG-DTA) to clarify the strength development of the mortar and cement-treated sand specimens. It should be noted that the compressive strengths of cement-treated sand reported in this study were partially adapted from the authors’ previous work [29,30]. This study is a completed investigation of that work, as it presents additional results pertaining to the compressive strength and TG-DTA findings. In particular, the relationships between the compressive strength and maturity index, and between the compressive strength and chemically bound water, are clarified as universal indices that can explain the properties of cement-treated sand and mortar in this study.

## 2. Materials and Measurements

### 2.1. Materials and Specimens

#### 2.1.1. Mix Proportions, Mixing and Compaction

Various specimens were created from cement-treated sand and mortar mixtures with OPC, HPC and MPC, to investigate the effects of cement type on strength development. Two cement-treated sand mixtures were prepared with different cement types at low contents (~8% and 15% of the mass of dried sand), and at a constant water-to-cement ratio (W/C) of 100%. Additionally, two mortar mixtures with respective W/C values of 50% and 100% were prepared with cement contents of 50% and 25%, respectively, of the mass of dried sand for each cement type. A mortar with a W/C of 50% was prepared to reflect a standard mortar used in concrete engineering, and a mortar with a W/C of 100% was produced to provide a high-porosity cement paste matrix similar to that of the cement-treated sand for comparison. Toyoura silica sand was used in this study as it is considered a standard sand type with low pozzolanicity, and is used extensively in experiments in Japan [31]. The particle size distribution of Toyoura sand can be found in the literature [31]. The specific densities of OPC, HPC, MPC and the sand were 3.15, 3.12, 3.20 and 2.65 g/cm^3^, respectively. The chemical compositions of the cements and sand are listed in Table 1, and the mineral constituents of the cements are listed in Table 2. In particular, the cement clinker phases were calculated using Bogue’s equation, and the amount of CaCO_3_ was calculated by assuming that the loss upon ignition is the same as the quantity of decarbonized CaCO_3_ in the cement. The designed proportions of the cement-treated sand and mortar mixtures are summarized in Table 3.

Based on Table 3, the water contents of the 8% and 15% cement-treated sand mixtures were calculated to be approximately 7.41% and 13.04%, respectively. These water contents were approximately optimal for the compaction test of these cement-treated sand mixtures. To control the moisture content of the cement-treated sand specimens, water and soil were first mixed using a mixer until a homogenous mixture was obtained, and the cement was then added. This mixing process was described in detail in our previous works [31,32]. After mixing, cylindrical specimens with a diameter and height of 50 mm and 100 mm, respectively, were cast by compaction with a 1.5 kg rammer. Owing to the limited volume of the mixer bowl used, the specimens were cast in several batches; however, the homogeneity across all batches was verified by checking the specimen densities.

The mortar specimens were mixed according to the recommendations of the Japanese Industrial Standard (JIS) R 5201 [33]. Water and cement were machine-mixed at a low speed (140 revolutions per minute (rpm)) for 30 s. Subsequently, sand was added to the mixture and remixing was performed for 30 s at the same low speed. The mixture was then mixed for 30 s at a high speed (280 rpm), then mixed by hand for 30 s, and was then covered with a wet towel and rested for 90 s. Finally, the mixture was mixed for 60 s at the same high speed and then manually stirred ten times using a spoon. After completing the mixing procedure, 50 mm × 100 mm cylindrical specimens were cast in three layers by tapping each layer 15 times. To prevent segregation of the mortar with a W/C of 100% after casting, the mixture was stored and regularly mixed in a plastic bag for ~5–7 h before casting. All mixing procedures were conducted at a constant temperature of 20 °C.

#### 2.1.2. Curing Conditions

To examine the strength development that resulted from cement hydration, the specimens were cured in sealed conditions. Specifically, all specimens were cast into thin steel cylindrical molds and their upper surfaces were tightly covered using aluminum tape at 20 °C. The specimens were then immediately cured at different temperatures (at 20 °C in a control room or at 40 °C in a control chamber) for various lengths of time (1, 3, 7, 14, 28, 56 and 91 days). A constant elevated temperature of 40 °C was employed to simulate the temperature generated by cement hydration in the core of a deep mixed cement-treated soil column, or the temperature of cement-treated soil in tropical regions [5,6]. To confirm that carbonation and drying did not occur, all specimens were constantly weighed throughout the entire curing period. Furthermore, a thermal analysis was performed to verify that carbonation did not occur.

### 2.2. Measurements

#### 2.2.1. Unconfined Compressive Strength

After the specified curing times, the compressive strengths of the cement-treated sand and mortar specimens were determined by axial compression tests at a constant loading rate of 0.1 mm/min [32,34]. The compressive strengths at each age and curing condition are reported in this study as the mean value of the compressive strengths of three identical specimens.

#### 2.2.2. Thermal Analysis

The chemically bound water contents were determined by TG-DTA. The samples were collected with a disk slice cut from the center of each specimen after the compression test. This slice was promptly soaked in an acetone solution for more than 24 h to prevent the occurrence of additional chemical reactions. Vacuum desiccation was subsequently performed for more than 24 h to dry the disk slice sample, which was then milled into a fine powder with particle diameters smaller than 150 μm. Approximately 60 mg of the powdered sample was used for the TG-DTA by heating from 20 °C to 1000 °C. The powder sample for TG-DTA was collected carefully based on the golden rule of sampling to obtain homogeneity [35]. Furthermore, as stated in previous publications [36,37], for a fine powder that has the same size as cement particles, a few grams of the powder contain a very high number of particles that can help reduce sampling errors significantly. Details of the TG-DTA process can be found in previous reports [31,32]. It should be noted that a nitrogen flow was applied to prevent the occurrence of carbonation during the heating process.

## 3. Results and Discussion

### 3.1. Compressive Strength

This subsection describes and compares the results of the compressive strength tests of the mortar and cement-treated sand specimens to understand the effects of cement type and curing temperature on the strength development of cement-treated sand. Subsequently, the effects of cement content on the strength development at different conditions are clarified. Finally, the relationship between specimen age and compressive strength is established for both cement-treated sand and mortar.

#### 3.1.1. Compressive Strength of Mortar

**Figure 1:**
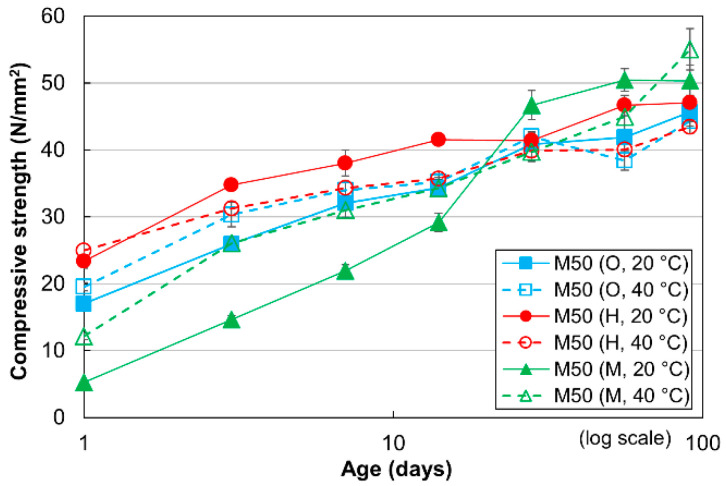
Compressive strengths of mortar with W/C values of 50% for three cement types at different curing temperatures.

Figure 1 shows the compressive strengths of mortar specimens with a W/C of 50% (standard mortar) for three different cement types cured at 20 °C and 40 °C. In general, the compressive strengths increase gradually as a function of the curing time from 1 to 91 days for all mortar specimens. This agrees well with the results obtained in previous studies [13,14,15,16,17,28,38,39,40]. The strengths of the OPC mortar specimens cured for 1 to 28 days at 40 °C are higher than those of the OPC mortar specimens cured at 20 °C. This phenomenon occurred because an elevated curing temperature promoted the cement hydration reaction, and resulted in an increase in the compressive strength. This tendency was also observed in previous studies on mortar and concrete [13,14,15,17]. In contrast, the compressive strengths of the OPC mortar specimens cured for 56 to 91 days are lower at 40 °C than at 20 °C owing to the cross-over effect, as reported in previous studies [16,17]. In the case of the HPC mortar specimens, the compressive strength at 40 °C is always less than that at 20 °C, except at the age of 1 day. In this case, the cross-over effect occurred earlier owing to the higher fineness of the HPC particles, which resulted in faster initial and final setting times. This finding was also consistent with the results of previous related studies [38,39]. At an age of 91 days, the compressive strengths of the OPC, HPC and MPC mortar specimens cured at 20 °C were nearly equivalent, as reported in previous studies [28,40]. However, the strength of the MPC mortar specimen cured at 40 °C was slightly higher than that of the OPC and HPC mortar specimens cured at 40 °C, as observed in a previous study [28].

**Figure 2:**
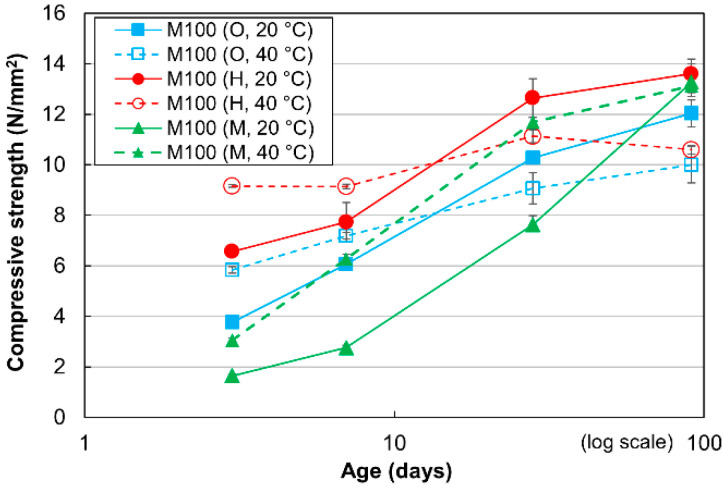
Compressive strengths of mortar with W/C values of 100% for three cement types at different curing temperatures.

The strength development of the mortar with a W/C of 100% (special mortar with high W/C) for the three different cement types cured at 20 °C and 40 °C is shown in Figure 2. Similar to the mortar with a W/C of 50%, the compressive strength gradually increases for curing times in the range of 1 to 91 days in all cases. The effects of the curing temperature were also similar: the strengths of all mortar specimens cured at 40 °C were higher than those cured at 20 °C up to 7 days of curing, while the opposite trend was observed from 28 to 91 days for both the OPC and HPC mortar specimens. In contrast to mortar specimens with W/C values equal to 50%, or for specimens with W/C values of 100% cured at 20 °C, the strengths of the HPC mortar specimens are always higher than those of the OPC and MPC specimens at all curing ages. However, when cured at 40 °C, the mortar specimens with W/C values of 100% are similar to those with W/C values of 50%; the HPC mortar specimen exhibits the lowest compressive strength at 91 days, because it shows a nearly constant strength following the rapid strength development for the first 3 days of curing.

#### 3.1.2. Compressive Strength of Cement-Treated Sand

**Figure 3:**
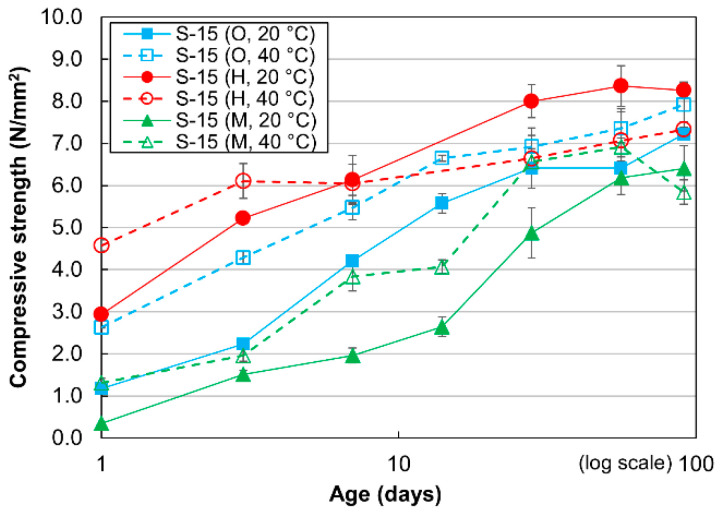
Compressive strengths of cement-treated sand mixture with 15% cement.

Figure 3 shows the compressive strength development of the cement-treated sand with 15% cement. In general, the compressive strength increases gradually from 1 to 91 days for all the cases, similar to the findings reported in studies pertaining to cement-treated clay [8,41]. In contrast to the mortar specimens, the compressive strengths of the OPC- and MPC-treated sand specimens cured at 40 °C are greater than the compressive strengths of those cured at 20 °C for all evaluated curing times. Similar to the equivalent mortar mixture, the compressive strengths of the HPC-treated sand specimens cured at 40 °C are higher than the compressive strengths of those cured at 20 °C up to 7 days of curing time, after which the trend reverses. This phenomenon occurred because the cement hydration was accelerated in the HPC-treated sand specimens, and thus led to an intense cross-over effect. Furthermore, when cured at 20 °C, the compressive strengths of the HPC-treated sand specimens were consistently greater than those of the OPC- and MPC-treated sand specimens, similar to the mortar specimens with W/C values of 100%, but in contrast to those with W/C values of 50%.

**Figure 4:**
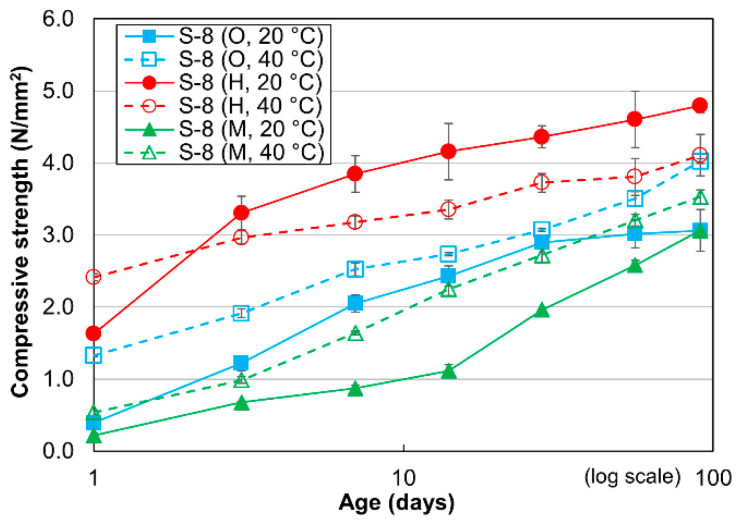
Compressive strengths of cement-treated sand mixture with 8% cement.

The compressive strengths of the cement-treated sand specimens with 8% cement and different cement types cured at 20 °C and 40 °C are shown in Figure 4. The compressive strengths gradually increase from 1 day to 91 days, as reported in previous studies on cement-treated clay [6,7,8,41,42] and similar to the other mixtures in this study. The compressive strengths of the OPC- and MPC-treated sand specimens are greater at 40 °C than at 20 °C for all evaluated curing times, similar to the cement-treated sand specimens with 15% cement shown in Figure 3 [8,41]. The compressive strengths of the HPC-treated sand specimens cured at 40 °C are consistently lower than the compressive strengths of those cured at 20 °C, with the exception of the responses generated after only 1 day of curing. This result is consistent with the mortars and cement-treated sand with 15% cement. Over the entire evaluated curing period, the HPC-treated sand specimens cured at 20 °C exhibited considerable compressive strengths compared with the other cases. In particular, the compressive strength of the HPC-treated sand specimen after 3 days of curing was slightly greater than that of the MPC-treated sand specimen after 91 days of curing at either temperature and that of the OPC-treated sand specimen cured at 20 °C, while it was slightly less than that of the OPC-treated sand specimen after 91 days of curing at 40 °C. The unique performance of the cement-treated sand with 8% cement is further clarified in Section 3.2.

#### 3.1.3. Effects of Cement Content on Strength Development under Different Curing Conditions

To examine the influences of the type of cement, cement content, and curing temperature on the strength development of the mortar and cement-treated sand specimens, the normalized compressive strength was established in terms of the cement content. Specifically, the normalized compressive strength was defined as the ratio of the compressive strength of a given specimen to that of the OPC specimen cured at 20 °C. The compressive strengths of specimens after curing for 3 days and 28 days were then compared to examine the strength development in early and later ages.

**Figure 5:**
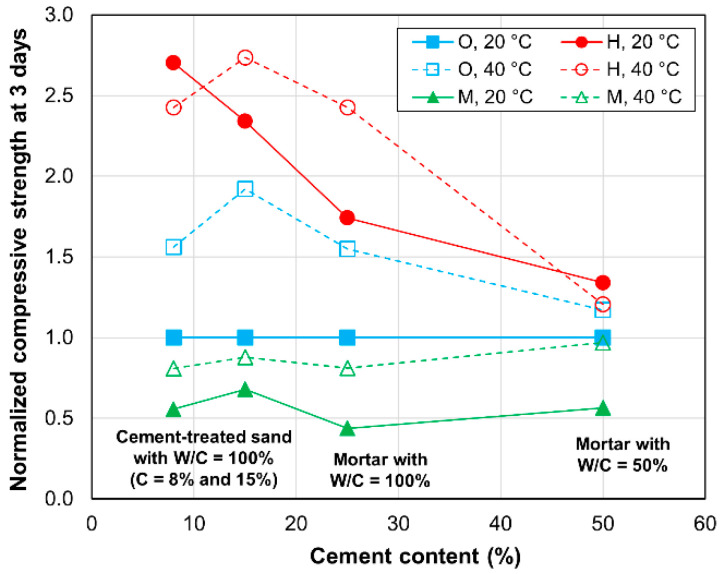
Normalized compressive strengths after 3 days of curing.

The normalized compressive strengths of the specimens cured for 3 days are shown in Figure 5 to exhibit the clear dependence of compressive strength on both cement type and curing temperature. In particular, the HPC specimens cured at any curing temperature and the OPC specimens cured at 40 °C have values greater than 1.0; however, the MPC specimens cured at any temperature have values that are smaller than 1.0. The HPC specimens consistently exhibit the highest values, highlighting the advantage of the use of HPC to promote early age strength development. Furthermore, deviations from 1.0 gradually increase with the specimens with cement contents of 50% (mortars with W/C values of 50%) to 8% (cement-treated sand). In particular, the normalized value for the HPC-treated sand specimens is approximately 2.5, which is nearly twice the corresponding value for the HPC mortar specimens with W/C values of 50%. Thus, the contribution of HPC to the early strength development of cement-treated sand can be considered more significant than its contribution to that of mortar.

**Figure 6:**
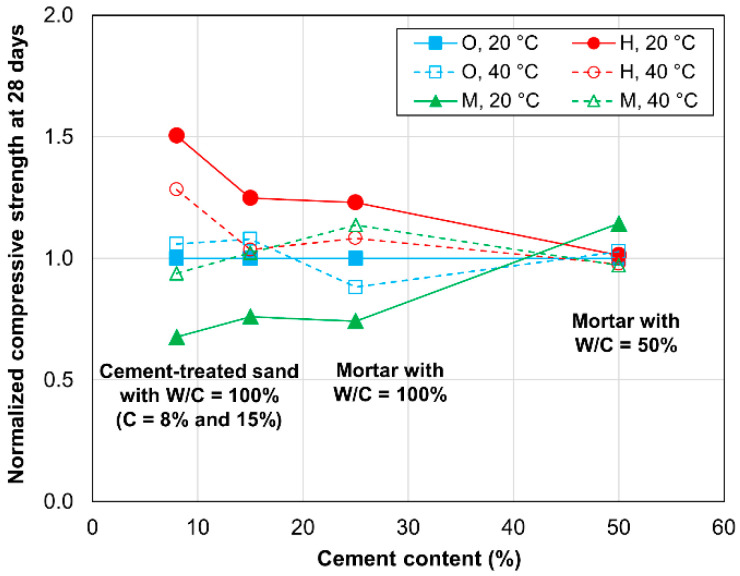
Normalized compressive strengths after 28 days of curing.

Figure 6 shows the normalized compressive strength of the specimens after curing for 28 days. Similar to the case associated with a curing time of 3 days, the normalized values are dependent on the cement type and curing temperature. Specifically, the values are larger than 1.0 for the HPC specimens cured at either temperature, with the exception of the mortar cases with W/C values of 50%. In contrast, the values were approximately equal to 1.0 for the OPC and MPC specimens cured at 40 °C, and were less than 1.0 for the MPC specimens cured at 20 °C. These results indicate the advantages associated with the use of HPC for increasing the compressive strength at 28 days. Furthermore, the normalized values of the HPC specimens cured at 40 °C are smaller than the normalized values of those cured at 20 °C, thus indicating that an elevated temperature negatively influences the strength development of both mortar and cement-treated sand that contains HPC. Overall, for the HPC specimens, the normalized values can be observed to gradually increase from those of the specimen with 50% cement (mortar with a W/C value of 50%) to those of the specimen with 8% cement (cement-treated sand). The normalized value is approximately 1.5 for cement-treated sand with 8% cement, or equivalently, is approximately 1.5 times higher than that of mortar with a W/C value of 50%. Therefore, the contribution of HPC to the strength development of cement-treated sand in later ages is still higher than its contribution to that of mortar, although the normalized values at 28 days are considerably smaller than those at 3 days. Thus, the contribution of HPC is more significant at early than at later ages for both cement-treated sand and mortar.

#### 3.1.4. Relationship between Compressive Strength and Maturity Index

The relationship between the compressive strength and maturity index is often used to illustrate the dependence of strength development in cement-treated sand and mortar on temperature. The maturity index is defined as follows [43,44].

(1) $$M=\sum \left(T-T_{0}\right)\times \Delta t$$

where *M* denotes the Nurse–Saul maturity index (°C·days), *T* is the mean temperature (°C) over the time interval Δt (days), and *T*_0_ indicates the datum temperature (°C).

**Figure 7:**
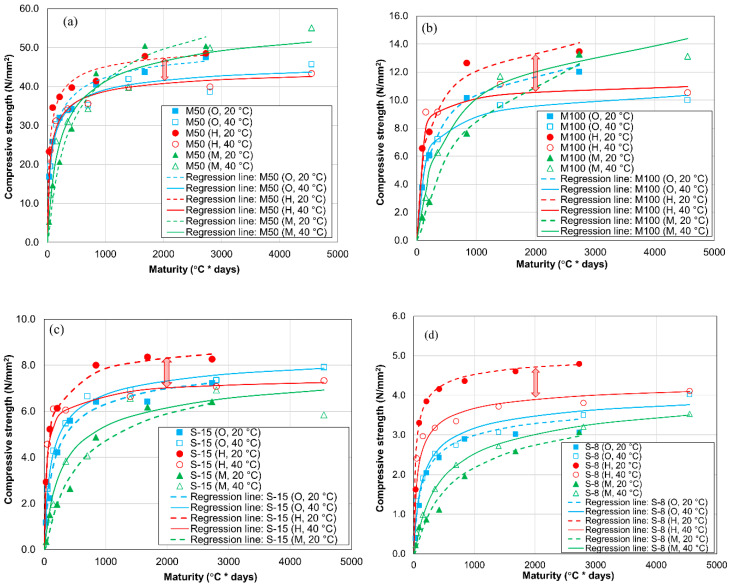
Strength–maturity relationship for the mortar and cement-treated sand specimens. (**a**) Mortar with a W/C value of 50%, (**b**) mortar with a W/C value of 100%, (**c**) cement-treated sand with 15% cement, and (**d**) cement-treated sand with 8% cement.

Figure 7 shows the relationship between the compressive strength and maturity index in the mortar and cement-treated sand specimens. The datum temperature is defined as the threshold temperature below which the strength will not increase. In this study, it was considered to be −10 °C [43,44,45]. In general, the differences between the OPC and MPC specimens cured at 20 °C and 40 °C are smaller than those between the HPC specimens cured at 20 °C and 40 °C. For the OPC and MPC specimens, the maturity index clearly describes the strength development of both mortar and cement-treated sand, as has been reported in previous studies on concrete cured at different temperatures [45,46]. However, in the case of the HPC mortar and cement-treated sand specimens, the strength development cannot be explained by the strength–maturity relationship, as a significant difference exists between the 20 °C and 40 °C curves; at the same maturity, a lower curing temperature corresponds to a higher compressive strength. This gap is likely related to the higher amount of alite present in HPC, which leads to faster hydration at a higher curing temperature, thereby adversely influencing the later strength through the cross-over effect. Furthermore, it has been reported that the maturity function cannot account for the detrimental impact of elevated curing temperature on the strength at the later age [45,47]. Therefore, although the maturity index is able to express the strength development of mortar and cement-treated sand specimens containing OPC and MPC, the strength development of HPC specimens cannot be described using this index.

### 3.2. Thermal Analysis

In this subsection, the method employed to determine the amount of chemically bound water is described first. Subsequently, the amounts of chemically bound water in the mortar and cement-treated sand specimens are presented and compared. Finally, the relationship between the compressive strength and amount of chemically bound water is established.

#### 3.2.1. Determination of Chemically Bound Water in Ground Cement Paste

In general, when a sample powder is heated from 100 °C to 1000 °C, the unhydrated minerals in the cement, as well as certain sand minerals, are decomposed. Therefore, the loss of the unhydrated cement minerals and sand minerals can be measured to calculate the amount of water in a sample that has been chemically bound by cement hydration. The amount of chemically bound water can be defined as follows.

(2) $$W_{CB}=\left(L_{100–1000}-L_{C:100–1000}\times \frac{C}{100}-L_{S:100–1000}\times \frac{S}{100}\right)\times \frac{100}{C}$$

where *W_CB_* is the amount of chemically bound water in the sample compared with the mass of cement (mass %), *C* and *S* denote the amount of cement and sand in the sample (mass%), respectively, *L*_100–1000_ denotes the mass loss in the sample from 100 to 1000 °C (mass%), *L*_*C*:100–1000_ denotes the mass loss in the unhydrated cement from 100 to 1000 °C (mass%), and *L*_*S*:100–1000_ denotes the mass loss in the sand minerals from 100 to 1000 °C (mass%).

#### 3.2.2. Amount of Chemically Bound Water (*W_CB_*)

**Figure 8:**
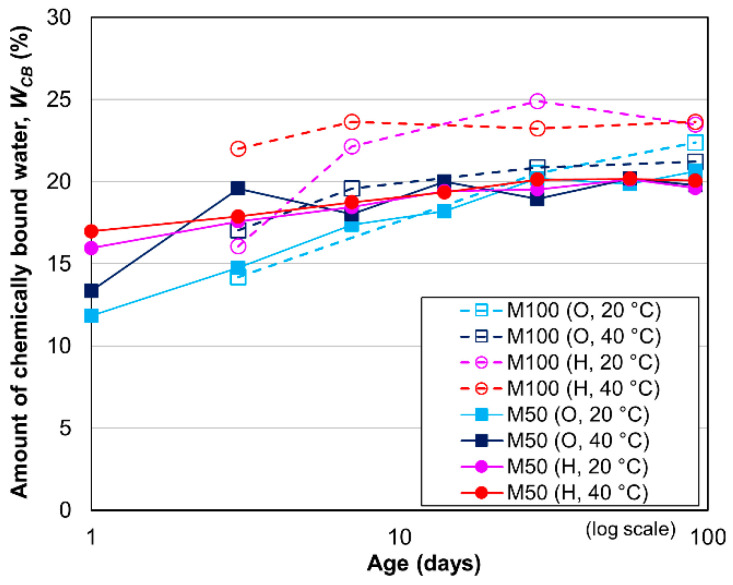
Amount of chemically bound water in mortars with W/C values of 50% and 100%.

Figure 8 shows the *W_CB_* in the ground cement pastes of the HPC and OPC mortars with W/C values of 50% and 100%. In all mortar specimens, the *W_CB_* increases gradually up to 14 days, after which it remains nearly constant, or increases slightly after 28 days. Figure 8 also indicates that the *W_CB_* values in the HPC and OPC mortars with W/C values of 50% are nearly equivalent. However, the *W_CB_* of the HPC mortar with a W/C value of 100% is larger than that of the equivalent OPC mortar. This larger value can be explained by the combined effect of the higher alite content in HPC (see Table 2) and the higher W/C ratio. As reported in a previous study, the hydration degree of alite is higher than that of belite, and a higher porosity with a larger W/C ratio leads to a greater degree of hydration of alite and belite [40].

**Figure 9:**
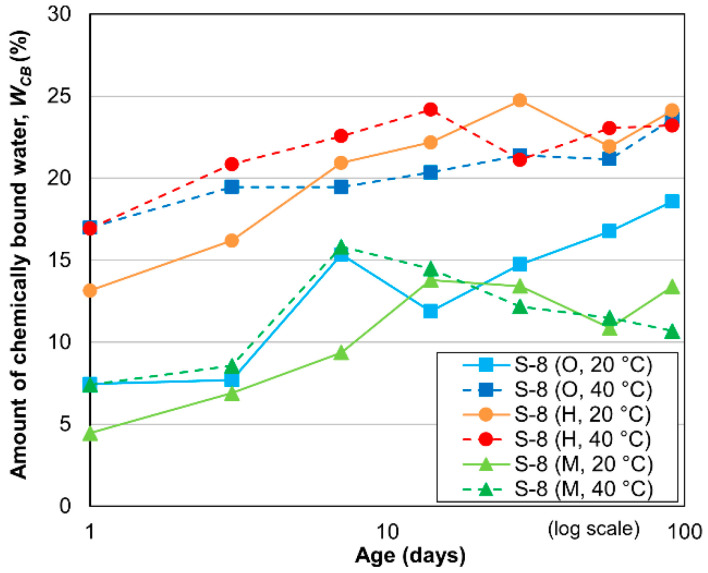
Amount of chemically bound water in cement-treated sands with 8% cement.

The change in the *W_CB_* of the ground cement paste of the cement-treated sand specimen with 8% cement with respect to time is shown in Figure 9. The *W_CB_* increases gradually from 1 to 14 days, after which it increases only slightly, as observed in the case of the mortar. Similar to the case of the mortar with a W/C value of 100%, the *W_CB_* values of the HPC-treated sand specimens cured at either temperature and of the OPC-treated sand specimen cured at 40 °C are considerably greater than those of the MPC-treated sand specimens cured at any temperature, or the OPC-treated sand specimen cured at 20 °C. This phenomenon occurred because of the larger amount of alite in HPC than in MPC, and this leads to faster and more complete hydration [40]. The difference in the cement mineral contents at elevated temperatures can thus result in different *W_CB_* values. The *W_CB_* values are the smallest and largest for the MPC- and HPC-treated sand specimens, respectively. Compared with the mortar specimens shown in Figure 8, a large gap is present between the *W_CB_* values of the HPC- and OPC-treated sand specimens cured at 20 °C. These results indicate that the cement mineral contents and curing temperature considerably influence the hydration process of cement-treated sand, even though a similar influence on mortar cannot be definitively observed.

**Figure 10:**
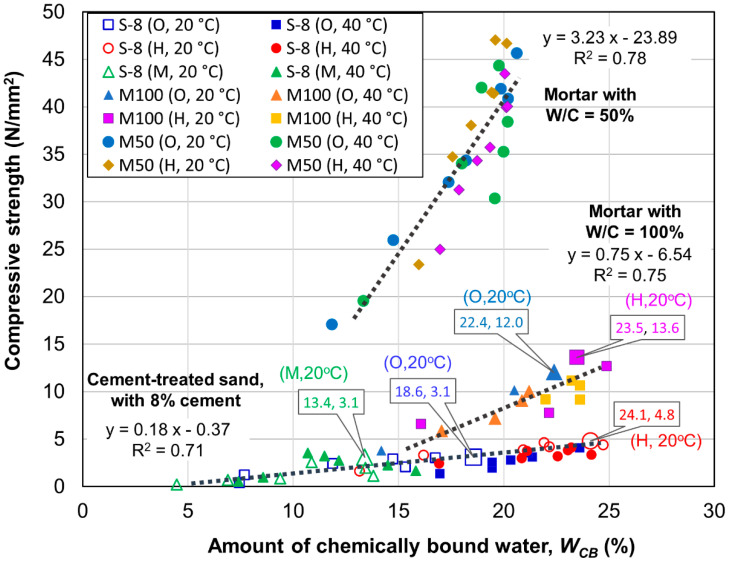
Relationship between strength and *W_CB_* for 8% cement-treated sand and mortar.

To explain the strength development of the cement-treated sand with 8% cement and compare the findings with those of mortar, the relationships between the compressive strength and *W_CB_* were established as shown in Figure 10. The compressive strength increases almost linearly as a function of *W_CB_* for all mixtures. The relationships do not appear to be significantly affected by cement type, whereas the gradients and maximum points of the linear relationships are strongly dependent on the mixture type in terms of cement contents (standard mortar, high-porosity mortar, or cement-treated sand). The gradients of these relationships decrease as the cement content decreases in the mixtures, with values of 3.23, 0.75 and 0.18 N/mm^2^/% for standard mortar, high-porosity mortar and cement-treated sand, respectively. This suggests that dense cement hydrates make a significant contribution to strength development. The maximum value of *W_CB_* is nearly equivalent (approximately 20%) for all mortar specimens with W/C values of 50%, thus indicating that the compressive strength should be nearly equivalent for these mixtures. This aspect helps explain the similar final compressive strengths obtained for the mortar specimens with W/C values of 50%. However, the mortar specimens with W/C values of 100% and cement-treated sand specimens exhibit shallower trends in their relationships between *W_CB_* and compressive strength in Figure 10, as the maximum *W_CB_* values for these mixtures were notably different for different cement types. At an age of 91 days (the maximum age for measurements in this study), the *W_CB_* values of the OPC and HPC mortar specimens with W/C = 100% cured at 20 °C were 22.4% and 23.5%, respectively. In the case of the cement-treated sand, the *W_CB_* values for the MPC, OPC and HPC specimens were 13.4%, 18.6% and 24.1%, respectively. The *W_CB_* values of the HPC-treated sand specimen are notably higher than those of the OPC- and MPC-treated sand specimens in Figure 10, and thus lead to a higher compressive strength.

## 4. Conclusions

In this study, the strength development of cement-treated sand was investigated with different cement types (HPC, OPC, and MPC) cured at different temperatures (20 °C and 40 °C), and the findings were compared with those obtained from mortars prepared under the same conditions. The following conclusions can be derived from the experimental results:

- An elevated curing temperature accelerated the early age strength development of both cement-treated sand and mortar specimens that contained HPC, OPC and MPC. However, the effect of temperature on the development of strength at the later age depended on the cement material used. Specifically, a higher temperature reduced the strength of the HPC mortar and cement-treated sand specimens, as well as that of the OPC mortar specimens, whereas it enhanced the strength of the OPC and MPC cement-treated sand specimens.
- For the HPC specimens, the normalized compressive strengths increased as the cement content decreased between mortars with W/C values of 50% and cement-treated sand with 8% cement. At the age of 3 days, the normalized value for the HPC-treated sand specimens is nearly twice the corresponding value for the HPC mortar specimens with W/C values of 50%. At the age of 28 days, the normalized value for cement-treated sand with 8% cement is approximately 1.5 times higher than that of mortar with a W/C value of 50%. The normalized values at an early age were greater than those at a later age. These findings indicated that the contributions of HPC were more significant for cement-treated sand than for mortar, and had a greater effect on the early age than the later-age strength.
- The maturity index expressed accurately the strength development of the OPC and MPC mortar and cement-treated sand specimens. However, the strength development of the HPC specimens could not be expressed with this index for either mortar or cement-treated sand.
- The higher compressive strength of the HPC-treated sand can be explained by the higher quantity of chemically bound water (24.1% at 91 days) owing to the higher alite content, higher water-to-cement ratio, and higher porosity of the specimens compared with those (13.4% and 18.6% at 91 days) of the OPC- and MPC-treated sand.

The findings of this study suggest that HPC can be effectively used as a novel method to provide a high internal temperature to accelerate the strength development of cement-treated sand cured at either elevated or normal temperature conditions. These findings improve our understanding of the strength development of both cement-treated sand and mortar based on considerations of the influence of the curing temperature and cement type. However, it should be noted that in this study, sand with a low pozzolanic activity was exclusively used. To determine the effects of the cement type (such as HPC) and the curing temperature on cement-treated soils more conclusively, it is necessary to extend this study to clays that exhibit high-pozzolanic activity.

## Figures and Tables

**Table 1:** Chemical compositions of sand, ordinary Portland cement (OPC), high early-strength Portland cement (HPC), and moderate heat Portland cement (MPC) (%).

Chemical Composition	OPC [31]	HPC	MPC	Toyoura Silica Sand [31]
Loss on ignition (LOI)	2.59	1.57	0.98	0.37
SiO_2_	20.18	20.19	23.60	92.54
Al_2_O_3_	5.19	4.99	3.73	2.96
Fe_2_O_3_	2.89	2.84	4.66	0.35
CaO	64.65	65.26	63.11	0.19
MgO	0.92	0.83	0.76	0.05
SO_3_	2.10	3.03	2.06	0.02
Na_2_O	0.24	0.20	0.23	0.58
K_2_O	0.40	0.34	0.23	2.75
Other	0.84	0.75	0.64	0.19

**Table 2:** Calculated mineral constituents of different cement types.

Cement Type	Calculated Mineral Component (Mass%)						
	C_3_S	C_2_S	C_3_A	C_4_AF	CaSO_4_	CaCO_3_	Others
HPC	57.80	14.36	8.42	8.63	5.15	3.57	2.06
OPC	51.35	19.20	8.87	8.79	3.57	5.89	2.34
MPC	34.82	41.48	2.01	14.17	3.50	2.23	1.80

**Table 3:** Designed mix proportions of cement-treated sand and mortar.

Mixture	Mixture Notation	Sand Content (kg/m^3^)	Cement/Sand (wt.%)	Water/Cement (W/C) (wt.%)
Cement-treated sand with 8% cement	S-8 (cement type *, curing temperature **)	1450	8	100
Cement-treated sand with 15% cement	S-15 (cement type, curing temperature)	1423	15	100
Mortar (W/C) = 100%)	M100 (cement type, curing temperature)	1415	25	100
Mortar (W/C = 50%)	M50 (cement type, curing temperature)	1270	50	50

Note: * Cement types are indicated as O (OPC), H (HPC), or M (MPC); ** Curing temperatures are 20 °C or 40 °C.

## References

[1] Horpibulsuk S., Rachan R., Chinkulkijniwat A., Raksachon Y., Suddeepong A. (2010). Analysis of strength development in cement-stabilized silty clay from microstructural considerations. Constr. Build. Mater..

[2] Subramanian S., Khan Q., Ku T. (2020). Effect of sand on the stiffness characteristics of cement-stabilized clay. Constr. Build. Mater..

[3] Kang G., Tsuchida T., Athapaththu A. (2016). Engineering behavior of cement-treated marine dredged clay during early and later stages of curing. Eng. Geol..

[4] Kitazume M., Terashi M. (2013). The Deep Mixing Method.

[5] Omura T., Murata M., Hirai M. The influence of strength due to hydration heat on-site measurement results and curing temperature of deep mixing. Proceedings of the 36th Japan Society of Civil Engineers Annual Meeting.

[6] Zhang R.J., Lu Y.T., Tan T.S., Phoon K.K., Santoso A.M. (2014). Long-term effect of curing temperature on the strength behavior of cement-stabilized clay. J. Geotech. Geoenviron. Eng..

[7] Lemaire K., Deneele D., Bonnet S., Legret M. (2013). Effects of lime and cement treatment on the physicochemical, microstructural and mechanical characteristics of a plastic silt. Eng. Geol..

[8] Wang D., Zentar R., Abriak N.E. (2016). Temperature-accelerated strength development in stabilized marine soils as road construction materials. J. Mater. Civ. Eng..

[9] Arabi M., Wild S. (1986). Microstructural development in cured soil-lime composites. J. Mater. Sci..

[10] Chew S.H., Kamruzzaman A.H.M., Lee F.H. (2004). Physicochemical and engineering behavior of cement treated clays. J. Geotech. Geoenviron. Eng..

[11] Horpibulsuk S., Rachan R., Suddeepong A. (2011). Assessment of strength development in blended cement admixed Bangkok clay. Constr. Build. Mater..

[12] Nakarai K., Yoshida T. (2015). Effect of carbonation on strength development of cement-treated Toyoura silica sand. Soils Found..

[13] Escalante-Garcıa J.I., Sharp J.H. (2001). The microstructure and mechanical properties of blended cements hydrated at various temperatures. Cem. Concr. Res..

[14] Brooks J.J., Al-Kaisi A.F. (1990). Early strength development of Portland and slag cement concretes cured at elevated temperatures. ACI Mater. J..

[15] Kim J.K., Han S.H., Song Y.C. (2002). Effect of temperature and aging on the mechanical properties of concrete: Part I. Experimental results. Cem. Concr. Res..

[16] Kim J.K., Moon Y.H., Eo S.H. (1998). Compressive strength development of concrete with different curing time and temperature. Cem. Concr. Res..

[17] Ezziane K., Bougara A., Kadri A., Khelafi H., Kadri E. (2007). Compressive strength of mortar containing natural pozzolan under various curing temperature. Cem. Concr. Compos..

[18] Carino N.J. (1984). The maturity method: Theory and application. Cem. Concr. Aggr. J..

[19] Mirza W.H., Al-Noury S.I., Al-Bedawi W.H. (1991). Temperature effect on strength of mortars and concrete containing blended cements. Cem. Concr. Compos..

[20] Ma W., Sample D., Martin R., Brown P.W. (1994). Calorimetric study of cement blends containing fly ash, silica fume, and slag at elevated temperatures. Cem. Concr. Aggr. J..

[21] Monzo J., Paya J., Peris-Mora E., Borrachero M.V. (1995). Mechanical treatment of fly ashes: Strength development and workability of mortars containing ground fly ashes. Spec. Publ..

[22] Videla C.C., Covarrubias J.P.T., Pascual J.M.D. (1996). Behaviour in extreme climates of concrete made with different types of cement. Concr. Serv. Mank. Appropr. Concr. Technol..

[23] Xiao R., Polaczyk P., Zhang M., Jiang X., Zhang Y., Huang B., Hu W. (2020). Evaluation of glass powder-based geopolymer stabilized road bases containing recycled waste glass aggregate. Transp. Res. Rec..

[24] Kürklü G. (2016). The effect of high temperature on the design of blast furnace slag and coarse fly ash-based geopolymer mortar. Compos. B Eng..

[25] Zhang P., Zheng Y., Wang K., Zhang J. (2018). A review on properties of fresh and hardened geopolymer mortar. Compos. B Eng..

[26] Wang X.Y. (2013). Simulation of temperature rises in hardening Portland cement concrete and fly ash blended concrete. Mag. Concr. Res..

[27] Sui T., Fan L., Wen Z., Wang J. (2015). Properties of Belite-Rich Portland Cement and Concrete in China. J. Civ. Eng. Arch..

[28] Sui T., Fan L., Wen Z., Wang J., Zhang Z. (2004). Study on the properties of high strength concrete using high belite cement. J. Adv. Concr. Technol..

[29] Nakarai K., Eguchi K., Ho L.S., Sasaki T. Effects of curing temperature and cement type on the reaction and strength development of cement-treated sand. Proceedings of the 71st Annual Meeting of Cement and Concrete Engineering.

[30] Ho L.S., Nakarai K., Eguchi K., Sasaki T., Morioka M. (2018). Strength development of cement-treated sand using different cement types cured at different temperatures. MATEC Web of Conferences.

[31] Ho L.S., Nakarai K., Duc M., Le Kouby A., Maachi A., Sasaki T. (2018). Analysis of strength development in cement-treated soils under different curing conditions through microstructural and chemical investigations. Constr. Build. Mater..

[32] Ho L.S., Nakarai K., Ogawa Y., Sasaki T., Morioka M. (2017). Strength development of cement-treated soils: Effects of water content, carbonation, and pozzolanic reaction under drying curing condition. Constr. Build. Mater..

[33] (2015). Physical testing methods for cement. Japan Industrial Standard (JIS).

[34] Okyay U.S., Dias D. (2010). Use of lime and cement treated soils as pile supported load transfer platform. Eng. Geol..

[35] Allen T. (2013). Particle Size Measurement.

[36] Scrivener K., Snellings R., Lothenbach B. (2018). A Practical Guide to Microstructural Analysis of Cementitious Materials.

[37] Ho L.S., Nakarai K., Ogawa Y., Sasaki T., Morioka M. (2018). Effect of internal water content on carbonation progress in cement-treated sand and effect of carbonation on compressive strength. Cem. Concr. Compos..

[38] Yousuf S., Shafigh P., Ibrahim Z., Hashim H., Panjehpour M. (2019). Crossover Effect in Cement-Based Materials: A Review. Appl. Sci..

[39] Wade S.A., Nixon J.M., Schindler A.K., Barnes R.W. (2010). Effect of temperature on the setting behavior of concrete. J. Mater. Civ. Eng..

[40] Maruyama I., Igarashi G. (2014). Cement reaction and resultant physical properties of cement paste. J. Adv. Concr. Technol..

[41] Bell F.G. (1996). Lime stabilization of clay minerals and soils. Eng. Geol..

[42] Al-Mukhtar M., Lasledj A., Alcover J.F. (2010). Behaviour and mineralogy changes in lime-treated expansive soil at 20 C. Appl. Clay Sci..

[43] Jin N.J., Seung I., Choi Y.S., Yeon J. (2017). Prediction of early-age compressive strength of epoxy resin concrete using the maturity method. Constr. Build. Mater..

[44] Soutsos M.N., Turu’allo G., Owens K., Kwasny J., Barnett S.J., Basheer P.A.M. (2013). Maturity testing of lightweight self-compacting and vibrated concretes. Constr. Build. Mater..

[45] Soutsos M., Hatzitheodorou A., Kanavaris F., Kwasny J. (2017). Effect of temperature on the strength development of mortar mixes with GGBS and fly ash. Mag. Concr. Res..

[46] Yikici T.A., Chen H.L.R. (2015). Use of maturity method to estimate compressive strength of mass concrete. Constr. Build. Mater..

[47] Soutsos M., Kanavaris F., Hatzitheodorou A. (2018). Critical analysis of strength estimates from maturity functions. Case Stud. Constr. Mater..

